# Associations of dietary diversity trajectories with depressive symptoms among Chinese older adults: a 10-year longitudinal study

**DOI:** 10.3389/fnut.2026.1873667

**Published:** 2026-08-21

**Authors:** Jiafa Yang, Yuxia Xie, Fengting Hu, Jin Peng, Weiran Wang, Lin Shen

**Affiliations:** 1 Faculty for Physical Education, Zhejiang Yuexiu University, Shaoxing, China; 2 Department of Arts and Sports, Dong-A University, Busan, Republic of Korea; 3 Faculty of Physical Activity and Sport Sciences, Polytechnic University of Madrid, Madrid, Spain

**Keywords:** depressive symptoms, dietary diversity, dose–response relationship, older adults, oral health, trajectory

## Abstract

**Background:**

Depression is one of the most prevalent mental health conditions among older adults in China, profoundly impairing quality of life and impeding healthy aging. Dietary diversity, as a comprehensive indicator of overall diet quality, has been associated with various chronic diseases and mental health outcomes. However, the association between longitudinal trajectories of the Dietary Diversity Score (DDS) and depressive symptoms among older adults remains poorly understood.

**Methods:**

Using four waves of longitudinal data from the Chinese Longitudinal Healthy Longevity Survey (CLHLS) spanning 2008 to 2018, a total of 1,538 participants aged ≥65 years were included. Latent Class Growth Analysis (LCGA) was applied to identify distinct long-term DDS trajectories, with CESD-10 depressive scores assessed in 2018 serving as the primary outcome. Three multivariable linear regression models examined the associations between DDS trajectories and depressive scores with stepwise covariate adjustment. Binary logistic regression sensitivity analyses, subgroup analyses, and Restricted Cubic Spline (RCS) analyses were conducted to examine the association between the number of teeth and depressive scores.

**Results:**

LCGA identified three DDS trajectories: stable (Class 1, *n* = 946), high-decreasing (Class 2, *n* = 205), and low-increasing (Class 3, *n* = 387). In the fully adjusted model, compared with Class 1, Class 2 had lower depressive scores (*B* = −0.902, 95% CI: −1.800 to −0.005), whereas Class 3 had higher depressive scores (*B* = 0.901, 95% CI: 0.200–1.602). Sensitivity analyses confirmed that Class 3 was associated with a higher risk of depressive symptoms (OR = 1.405, 95% CI: 1.064–1.857). RCS analyses revealed a nonlinear association between the number of teeth and depressive scores (*p* < 0.001), with predicted depressive scores showing a pronounced and sustained decline when the number of teeth exceeded 20.

**Conclusion:**

Long-term maintenance of higher dietary diversity was associated with lower depressive scores among Chinese older adults. A nonlinear dose–response relationship was observed between number of teeth and depressive symptoms. These findings suggest that maintaining greater dietary diversity in early aging, together with good oral health, higher health literacy, and better quality of life, may be associated with better mental health in older adults.

## 1 Introduction

The accelerating pace of global population aging has brought mental health issues among older adults into increasingly sharp focus. Depression is among the most prevalent psychiatric disorders in older populations, profoundly impairing daily functioning and overall physical and mental well-being (1). As the country with the largest aging population worldwide (2), China is home to approximately 190 million individuals aged 65 years and older, representing 13.5% of the total population (3). A systematic review and meta-analysis estimated that the overall prevalence of depressive symptoms among older adults in China is approximately one in five (4). Beyond its impact on individual health, depression accelerates the progression of chronic diseases, increases all-cause mortality, and imposes substantial social and healthcare economic burdens (5), rendering it a public health concern of considerable urgency.

The etiology of late-life depression is multifactorial, arising from the interplay of psychological, social, biological, and a range of other factors (6). Among the modifiable risk factors, diet has emerged as a potentially influential determinant. Compared with other lifestyle factors, dietary behavior is more amenable to modification and optimization, making it a promising entry point for chronic disease prevention and the promotion of healthy aging (7, 8). Diet may affect the onset and progression of depression through multiple physiological pathways, including the regulation of systemic inflammation (9), modulation of gut microbiota composition (10, 11), and influence on oxidative stress metabolism (12, 13). Among available dietary assessment tools, the Dietary Diversity Score (DDS) has been widely adopted due to its simplicity of administration and strong clinical relevance. Multiple studies have shown that DDS is an effective indicator of overall diet quality and nutritional adequacy (14, 15). Higher DDS has been consistently associated with reduced risks of multiple adverse health outcomes (16–18). In the domain of mental health, Li et al. (19) reported that higher DDS was associated with a lower risk of depressive symptoms among adults in the United States. A cross-sectional study of Spanish adults by Cano-Ibáñez et al. (20) similarly identified a significant inverse association between DDS and depressive symptoms.

Nevertheless, the majority of existing studies have employed cross-sectional designs, which are inherently unable to capture the long-term dynamic trajectory of DDS in older populations. In reality, individuals may follow markedly divergent trajectories of dietary diversity change across the aging process (21), and relying on a single time-point measurement of DDS is insufficient to elucidate its dynamic influence on depressive outcomes. Furthermore, tooth loss is a highly prevalent oral health condition in older adults that may directly constrain an individual’s capacity to consume a varied diet, thereby compromising dietary diversity (22) and potentially contributing to a higher risk of depressive symptoms (23). However, large-scale longitudinal evidence simultaneously examining the interrelationships among long-term DDS trajectories, number of teeth, and depressive symptoms remains scarce. Moreover, existing research has predominantly focused on populations with specific diseases or single-gender groups (24–27), with comparatively limited attention directed toward depression in older adults with general health profiles or comorbid chronic conditions. Studies specifically investigating the dose–response relationship between number of teeth and depressive symptoms in older adults are also notably lacking.

Against this background, the present study leverages a nationally representative ten-year longitudinal cohort dataset from China. Latent Class Growth Analysis (LCGA) was applied to identify distinct long-term DDS trajectories among older adults, and Restricted Cubic Spline (RCS) modeling was employed to examine the association between number of teeth and depressive scores. The study aimed to examine the associations between different DDS trajectories and depressive symptoms and to characterize the dose–response relationship between number of teeth and depressive scores. By adopting a dynamic trajectory perspective, this study provides longitudinal evidence on the relationships between dietary diversity trajectories, oral health, and depressive symptoms, with implications for dietary intervention and mental health promotion in older adults.

## 2 Methods

### 2.1 Study design and participants

**Figure 1:**
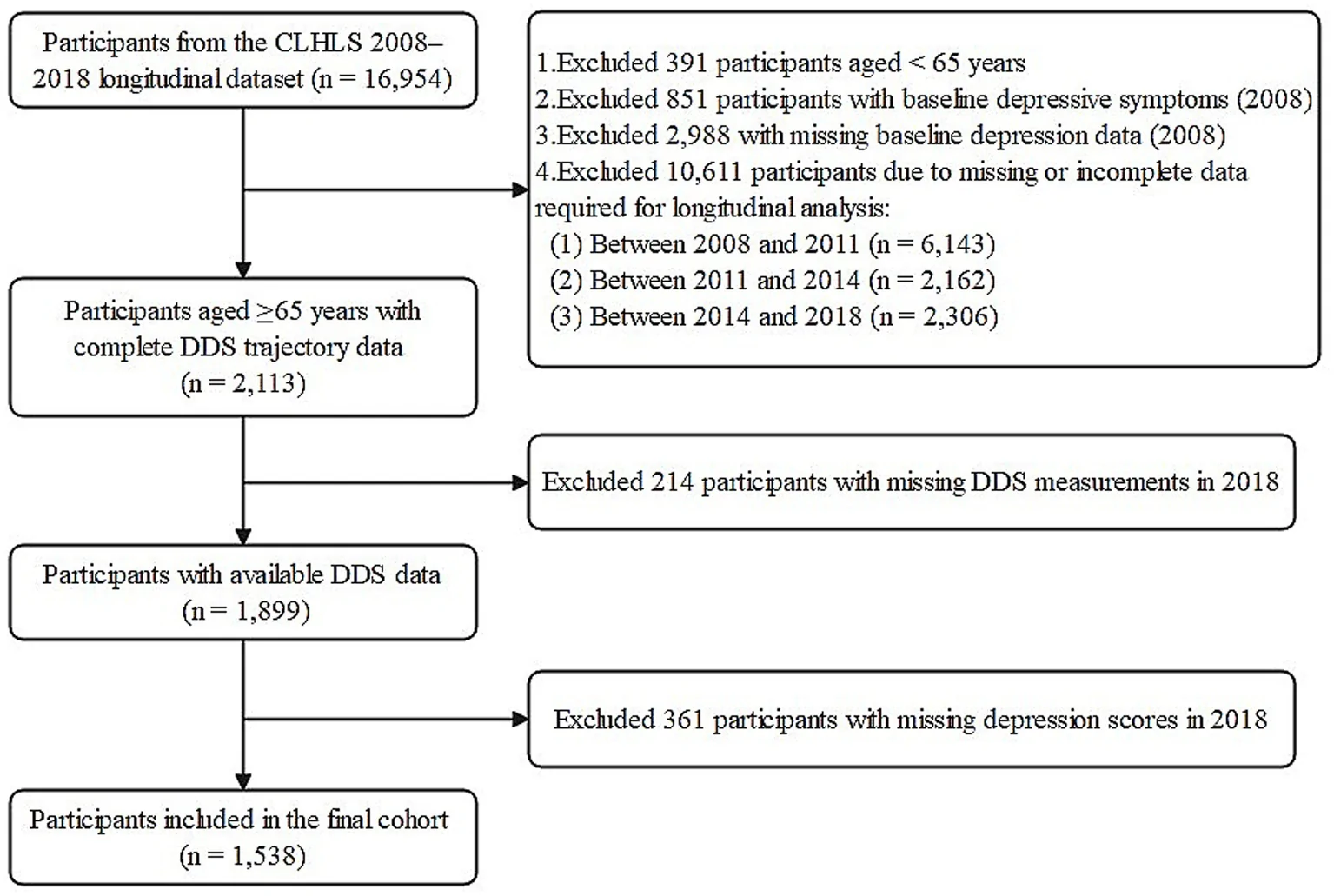
Flowchart of the screening process.

Data for this study were drawn from four waves of the Chinese Longitudinal Healthy Longevity Survey (CLHLS) conducted in 2008, 2011, 2014, and 2018, covering a 10-year follow-up period. Initiated in 1998, the CLHLS is a large-scale, nationally representative longitudinal survey covering 22 provinces, municipalities, and autonomous regions across China. The survey encompasses information on participants’ basic sociodemographic characteristics (including gender, marital status, household registration type, and education level), lifestyle behaviors, and health status. All participants provided written informed consent prior to enrollment, and the study protocol was approved by the Biomedical Ethics Committee of Peking University (IRB00001052-13074). Eligible participants were required to be aged ≥65 years at baseline (2008) and to have complete DDS data across all four survey waves. Exclusion criteria included a diagnosis of depression at baseline and missing data on key variables or covariates. Following the application of these criteria, a total of 1,538 participants were included in the final analytic sample. The detailed participant selection procedure is presented in Figure 1.

### 2.2 Measures

#### 2.2.1 Dietary diversity score

In this study, DDS was assessed across the 2008, 2011, 2014, and 2018 CLHLS survey waves using a food frequency questionnaire encompassing 13 food groups. This questionnaire has been widely used and validated in studies of older Chinese populations (21, 28). The 13 food groups included fresh fruit, vegetables, salt-preserved vegetables, sugar, meat, fish, eggs, beans, tea, garlic, dairy products, nut products, and mushrooms or algae. For fresh fruit and vegetables, a score of 1 was assigned if intake frequency was reported as “almost every day” or “except in winter,” and a score of 0 was assigned for “occasionally” or “rarely or never.” For the remaining food groups, a score of 1 was assigned for “almost every day” or “not every day but at least once per week,” while a score of 0 was assigned for “not every week but at least once per month,” “not every month but occasionally,” or “rarely or never.” Accordingly, the total DDS ranged from 0 to 13, with higher scores indicating greater dietary diversity.

#### 2.2.2 Depressive symptoms assessment

Depressive symptoms at baseline were assessed using five items from the 2008 CLHLS questionnaire, including questions such as “Do you always look on the bright side of things?,” “Do you feel lonely and isolated?,” and “Are you as happy as when you were younger?” Each item was rated on a five-point Likert scale ranging from 0 (always) to 4 (rarely or never), with “look on the bright side of things” and “are you as happy as when you were younger?” scored in reverse. Total scores ranged from 0 to 20, with lower scores indicating more severe depressive symptoms; a total score of ≤7 was used to identify participants with depressive symptoms at baseline and to apply the exclusion criterion (29). Depressive symptoms at follow-up were assessed using a standardized and validated scale, the Chinese version of the 10-item Center for Epidemiologic Studies Depression Scale (CESD-10), administered during the 2018 CLHLS survey (30). The CESD-10 comprises 10 items, each scored from 0 to 3. Three positively worded items (e.g., “Do you feel hopeful about the future?”) were reverse-scored, whereas the remaining seven items were scored in the standard direction. Total scores range from 0 to 30, with higher scores indicating greater severity of depressive symptoms. A total score of ≥10 was used to define the presence of depressive symptoms (31). The Chinese version of the CESD-10 has demonstrated good reliability and validity, and it has been widely used to assess depressive symptoms among older Chinese adults (31, 32). In the present study, the CESD-10 also demonstrated good internal consistency, with a Cronbach’s alpha coefficient of 0.825, further supporting its reliability in the present study population.

#### 2.2.3 Covariates

Covariates in this study were categorized into three domains. Sociodemographic characteristics included gender (male and female), household registration (urban and rural), education level (illiterate, primary school, and secondary school and above), and marital status (married, divorced/widowed, and never married). Lifestyle factors included smoking status (yes and no), alcohol consumption (yes and no), and physical activity (yes and no). Health-related variables included number of chronic diseases [0 (no chronic disease), 1 (single chronic disease), and ≥2 (multiple chronic conditions)], number of teeth (0–32 natural dentition), quality of life, and sleep quality. Chronic disease burden was derived from 22 chronic conditions documented in the 2008 CLHLS baseline survey, including conditions such as heart disease, hypertension, and diabetes. Quality of life was assessed using a single self-rated question: “How would you rate your quality of life?” with response options of very good, good, fair, poor, and very poor. Sleep quality was assessed using a single self-rated question: “How is your sleep quality?” with dichotomous response options of good and poor. All covariates were obtained from the 2008 baseline survey. These variables were treated as baseline characteristics in the regression models to account for participants’ initial sociodemographic, lifestyle, and health status.

These covariates were selected *a priori* based on previous literature demonstrating their potential associations with both dietary diversity and depressive symptoms in older adults (15, 20). Sociodemographic characteristics, lifestyle behaviors, and health-related conditions may influence dietary patterns and mental health outcomes and were therefore included as potential confounders in the multivariable analyses.

### 2.3 Statistical analysis

All statistical analyses were conducted using SPSS 28.0 and R 4.3.3. A two-sided significance threshold of *p* < 0.05 was applied throughout. Continuous variables are presented as mean ± standard deviation, and categorical variables as frequencies and percentages. Between-group comparisons for continuous variables were performed using one-way analysis of variance (ANOVA), and chi-square tests were used for categorical variables.

LCGA was applied to identify distinct DDS trajectories using DDS measurements obtained at four time points (2008, 2011, 2014, and 2018). The optimal number of latent trajectory classes was determined based on multiple model fit criteria, including the Akaike Information Criterion (AIC), Bayesian Information Criterion (BIC), entropy, average posterior probabilities, minimum class membership proportion, and clinical interpretability. Model adequacy required an average posterior probability exceeding 0.70 for each class and a minimum class membership proportion of 5% of the total sample. Models specifying one to five trajectory classes were evaluated. Based on the overall model fit and clinical interpretability, the three-class solution was selected, comprising Class 1 (stable), Class 2 (high-decreasing), and Class 3 (low-increasing).

The primary analysis employed three multivariable linear regression models to examine the associations between DDS trajectories and CESD-10 depressive scores in 2018. Using Class 1 as the reference group, Class 2 and Class 3 were entered as dummy variables. Model 1 adjusted for sociodemographic characteristics; Model 2 additionally incorporated lifestyle factors; and Model 3 further adjusted for health-related variables. Regression coefficients (B) and their corresponding 95% confidence intervals (CIs) were reported.

Sensitivity analyses were performed by dichotomizing the 2018 CESD-10 depressive score, with a score of ≥10 defined as the presence of depressive symptoms (32). Binary logistic regression was performed to re-examine the associations between DDS trajectories and depressive symptoms, adjusting for the same set of covariates. Effect sizes are reported as odds ratios (ORs) with 95% CIs. To explore potential heterogeneity in the observed associations, exploratory subgroup analyses were conducted according to participant characteristics, including gender, education level, marital status, household registration, physical activity, chronic disease status, sleep quality, and number of teeth. Within each subgroup, binary logistic regression was applied to examine the associations between DDS trajectories and depressive symptoms, with identical covariate adjustment.

RCS analysis was performed to characterize the dose–response relationship between number of teeth and depressive scores. Number of teeth was treated as a continuous variable within a multivariable linear regression framework. Following the recommendation of Harrell (33), four knots were specified at the 5th, 35th, 65th, and 95th percentiles. The same covariates were adjusted throughout, and a nonlinearity test was conducted to assess whether the dose–response relationship deviated significantly from linearity.

Prior to model estimation, the assumptions of multiple linear regression were evaluated. Multicollinearity was assessed using variance inflation factors (VIFs), while normality and homoscedasticity were examined through residual diagnostic plots. No substantial violations of model assumptions were detected.

## 3 Results

### 3.1 DDS trajectories

**Figure 2:**
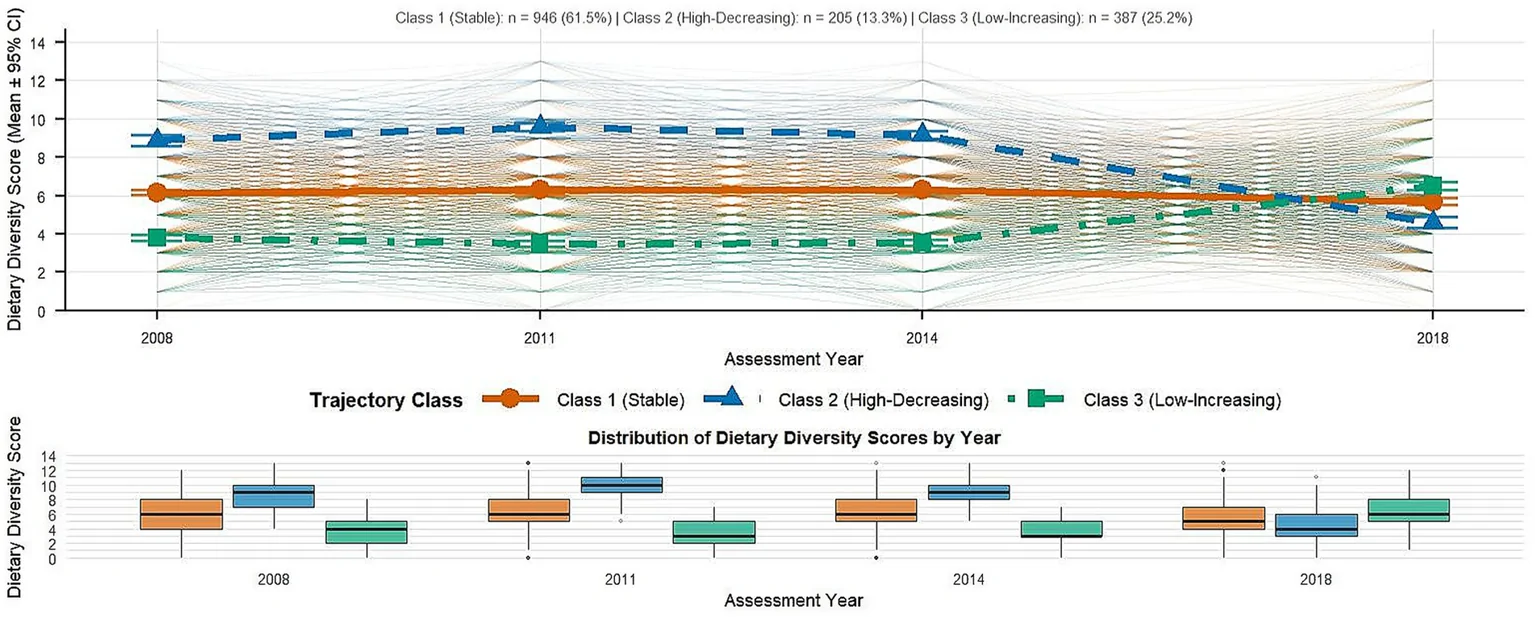
Trajectories of dietary diversity scores.

LCGA was applied to model the longitudinal trajectories of DDS. Model fit statistics for one- to five-class solutions are presented in Supplementary Table S1. The three-class model was selected based on a balance between statistical fit and clinical interpretability, including the lowest BIC, an acceptable entropy value (0.78), average posterior probabilities for the three classes ranging from 0.767 to 0.815, and all trajectory groups exceeding the recommended minimum class size of 5%. Class 1 represented the stable trajectory, characterized by a relatively consistent DDS over the ten-year observation period, with scores declining gradually from 6.16 in 2008 to 5.69 in 2018 (change: −0.47), comprising 946 participants (61.51%). Class 2 represented the high-decreasing trajectory, with the highest baseline DDS (8.88) among the three classes; scores declined sharply over time, reaching 4.58 by 2018 (change: −4.30), making this the trajectory with the most pronounced decline, comprising 205 participants (13.33%). Class 3 represented the low-increasing trajectory, characterized by the lowest baseline DDS (3.78) among the three classes, with scores rising progressively to 6.51 by 2018 (change: +2.74), comprising 387 participants (25.16%). The longitudinal trends of the three DDS trajectories are illustrated in Figure 2.

### 3.2 Characteristics of the study sample

A total of 1,538 older adults were included in the study. Detailed baseline characteristics stratified by DDS trajectory class are presented in Table 1. The mean age of the study sample was 73.95 years, with 759 (49.3%) males and 779 (50.7%) females. With respect to educational attainment, 43.6% were illiterate, 40.8% had completed primary school, and 15.6% had attained secondary school education or above. The majority of participants were married (65.5%), and 68.0% held urban household registration. Regarding health-related variables, the mean number of teeth was 14.27, the mean quality of life score was 2.37, and the mean depressive score was 12.04. Approximately 37.0% of participants engaged in regular physical activity, and 64.1% self-reported good sleep quality. Additionally, 48.2% of participants had one or more chronic diseases, though between-group differences in the number of chronic diseases did not reach statistical significance.

**Table 1:** Baseline characteristics of participants.

Characteristics	Class 1 (Stable) *N* = 946	Class 2 (High-decreasing) *N* = 205	Class 3 (Low-increasing) *N* = 387	Total *N* = 1,538	*p-*value
Age (years), mean ± SD	73.97 ± 7.11	72.60 ± 6.49	74.63 ± 7.53	73.95 ± 7.16	0.002
Gender, *n* (%)					<0.001
Male	491 (51.9%)	124 (60.5%)	144 (37.2%)	759 (49.3%)	
Female	455 (48.1%)	81 (39.5%)	243 (62.8%)	779 (50.7%)	
Household registration, *n* (%)					<0.001
Urban	646 (68.3%)	80 (39.0%)	320 (82.7%)	1,046(68.0%)	
Rural	300 (31.7%)	125 (61.0%)	67 (17.3%)	492 (32.0%)	
Education, *n* (%)					<0.001
Illiterate	410 (43.3%)	39 (19.0%)	221 (57.1%)	670 (43.6%)	
Primary school	394(41.7%)	94 (45.9%)	140 (36.2%)	628 (40.8%)	
Secondary school and above	142 (15.0%)	72 (35.1%)	26 (6.7%)	240 (15.6%)	
Marital status, *n* (%)					<0.001
Married	639 (67.5%)	157 (76.6%)	212 (54.8%)	1,008(65.5%)	
Divorced/widowed	297 (31.4%)	48 (23.4%)	170 (43.9%)	515 (33.5%)	
Never married	10 (1.1%)	0 (0%)	5 (1.3%)	15 (1.0%)	
Smoking, *n* (%)					0.105
Yes	236 (24.9%)	55 (26.8%)	78 (20.2%)	369 (24.0%)	
No	710 (75.1%)	150 (73.2%)	309 (79.8%)	1,169(76.0%)	
Alcohol consumption, *n* (%)					0.029
Yes	229 (24.2%)	59 (28.8%)	75 (19.4%)	363 (23.6%)	
No	717 (75.8%)	146 (71.2%)	312 (80.6%)	1,175(76.4%)	
Physical activity, *n* (%)					<0.001
Yes	361 (38.2%)	116 (56.6%)	92 (23.8%)	569 (37.0%)	
No	585 (61.8%)	89 (43.4%)	295 (76.2%)	969 (63.0%)	
Sleep quality, *n* (%)					<0.001
Good	630 (66.6%)	140 (68.3%)	216 (55.8%)	986 (64.1%)	
Poor	316 (33.4%)	65 (31.7%)	171 (44.2%)	552 (35.9%)	
Number of chronic diseases, *n* (%)					0.279
0 (No)	502 (53.1%)	105 (51.2%)	190 (49.1%)	797 (51.8%)	
1 (Single chronic disease)	283 (29.9%)	59 (28.8%)	136 (35.1%)	478 (31.1%)	
≥2(Multiple chronic conditions)	161 (17.0%)	41 (20.0%)	61 (15.8%)	263 (17.1%)	
Number of teeth, mean ± SD	15.92 ± 11.28	13.05 ± 10.07	14.41 ± 10.52	14.27 ± 10.54	0.016
Quality of life score, mean ± SD	2.05 ± 0.79	2.67 ± 0.82	2.31 ± 0.76	2.37 ± 0.80	<0.001
Depression score, mean ± SD	10.15 ± 5.77	13.50 ± 5.86	11.86 ± 5.80	12.04 ± 5.90	<0.001

p < 0.05 indicates statistically significant difference among the three groups.

### 3.3 Associations between DDS trajectories and depressive scores

Prior to model estimation, regression diagnostics were performed to evaluate the assumptions of multiple linear regression. Residual Q–Q plot indicated approximate normality of the residuals, with only minor deviations observed at the distribution tails (Supplementary Figure S1). Examination of residual diagnostic plots revealed no substantial heteroscedasticity. In addition, all VIF were below 1.3, indicating negligible multicollinearity among the covariates (Supplementary Tables S2, S3). These findings support the validity of the regression models.

To examine the independent associations between DDS trajectories and depressive scores, multivariable linear regression analyses were conducted with stepwise covariate adjustment across three sequentially constructed models. Results are presented in Table 2. Model 1 included DDS trajectory variables and sociodemographic characteristics. Compared with Class 1, Class 2 exhibited significantly lower depressive scores (*B* = −1.055, 95% CI: −1.960 to −0.150, *p* = 0.022), while Class 3 demonstrated significantly higher depressive scores (*B* = 1.250, 95% CI: 0.554 to 1.946, *p* < 0.001). Model 2 additionally adjusted for lifestyle factors, and the associations of DDS trajectories with depressive scores remained statistically significant: Class 2 (*B* = −1.048, 95% CI: −1.954 to −0.142, *p* = 0.023) and Class 3 (*B* = 1.249, 95% CI: 0.551 to 1.947, *p* < 0.001). No significant associations were observed between lifestyle factors and depressive scores (all *p* > 0.05). Model 3 further adjusted for health-related variables, including number of chronic diseases, sleep quality, number of teeth, and quality of life score. The associations between DDS trajectories and depressive scores remained statistically significant: compared with Class 1, Class 2 continued to exhibit significantly lower depressive scores (*B* = −0.902, 95% CI: −1.800 to −0.005, *p* = 0.049), and Class 3 continued to demonstrate significantly higher depressive scores (*B* = 0.901, 95% CI: 0.200 to 1.602, *p* = 0.011). Primary school and secondary school or above education levels, as well as good sleep quality, were associated with lower depressive scores compared with their respective reference groups. Number of teeth was significantly and inversely associated with depressive scores. Quality of life score was significantly and positively associated with depressive scores; as the scale was reverse-coded, higher scores reflected poorer quality of life. Furthermore, having two or more chronic diseases showed a marginally significant positive association with depressive scores (*B* = 0.700, 95% CI: −0.115 to 1.515, *p* = 0.092).

**Table 2:** Multiple linear regression of DDS trajectories and depressive scores.

Variables	Ref	Model 1	Model 2	Model 3
DDS trajectory	Class1			
Class2 (High-decreasing)		−1.055 (−1.960, −0.150)^*^	−1.048 (−1.954,-0.142)^*^	−0.902 (−1.800,-0.005)^*^
Class3 (Low-increasing)		1.250 (0.554,1.946)^***^	1.249 (0.551,1.947)^***^	0.901 (0.200, 1.602)^*^
Gender	Female			
Male		−0.464 (−1.116, 0.188)	−0.160 (−0.894,0.573)	0.102 (−0.627, 0.832)
Household registration	Rural			
Urban		0.370 (−0.281, 1.022)	0.399 (−0.279,1.078)	0.366 (−0.305, 1.037)
Marital status	Married			
Divorced/ widowed		0.284 (−0.368, 0.937)	0.303 (−0.350,0.956)	0.166 (−0.485, 0.817)
Never married		3.638 (0.694, 6.583)*	3.615 (0.668,6.563)^*^	3.241 (0.330, 6.152)^*^
Education	Illiterate			
Primary school and above		−1.158(−1.826, −0.490)^**^	−1.134 (−1.803, −0.464)^**^	−1.132 (−1.797, −0.468)^**^
Secondary school and above		−1.955(−2.904,-1.006)^***^	−1.968 (−2.921,-1.015)^***^	−1.835 (−2.789, −0.882)^***^
Physical activity	No			
Yes			0.005 (−0.635, 0.645)	0.020 (−0.617, 0.657)
Alcohol consumption	No			
Yes			−0.234 (−0.979, 0.512)	−0.120 (−0.857, 0.617)
Smoking	No			
Yes			−0.609 (−1.375, 0.157)	−0.805 (−1.564, −0.046)^*^
Number of chronic diseases	0			
1				0.510 (−0.146, 1.166)
≥2				0.700 (−0.115, 1.515)
Sleep quality	Poor			
Good				−1.127 (−1.748, −0.505)^***^
Number of teeth (continuous)				−0.035 (−0.063,-0.007)^*^
Quality of life (continuous)				0.650 (0.274,1.025)^**^
Constant		12.123^***^	12.109^***^	11.557^***^
R^2^		0.055	0.057	0.086

*P < 0.05;**P < 0.01; ***P < 0.001; CI, confidence interval.

### 3.4 Sensitivity analyses

Sensitivity analyses were conducted by dichotomizing the 2018 depressive score, using the same covariate adjustment strategy as in the primary analysis (Supplementary Table S4). Compared with Class 1, the association between Class 2 and depressive symptoms did not reach statistical significance (OR = 0.784, 95% CI: 0.566–1.085, *p* = 0.143); however, the OR was less than 1, indicating a protective direction consistent with the primary analysis. In contrast, Class 3 remained significantly associated with a higher risk of depressive symptoms (OR = 1.405, 95% CI: 1.064–1.857, *p* = 0.017), also consistent with the primary analysis. In the fully adjusted model, secondary school education or above was associated with a stronger protective effect (OR = 0.523, 95% CI: 0.368–0.743, *p* < 0.001). Each additional tooth was associated with an approximately 1.4% reduction in depression risk (OR = 0.986, 95% CI: 0.976–0.996, *p* = 0.008). Compared with participants without chronic diseases, those with ≥2 chronic conditions exhibited a significantly higher risk of depressive symptoms (OR = 1.386, 95% CI: 1.011–1.900, *p* = 0.042).

### 3.5 Subgroup analyses

**Figure 3:**
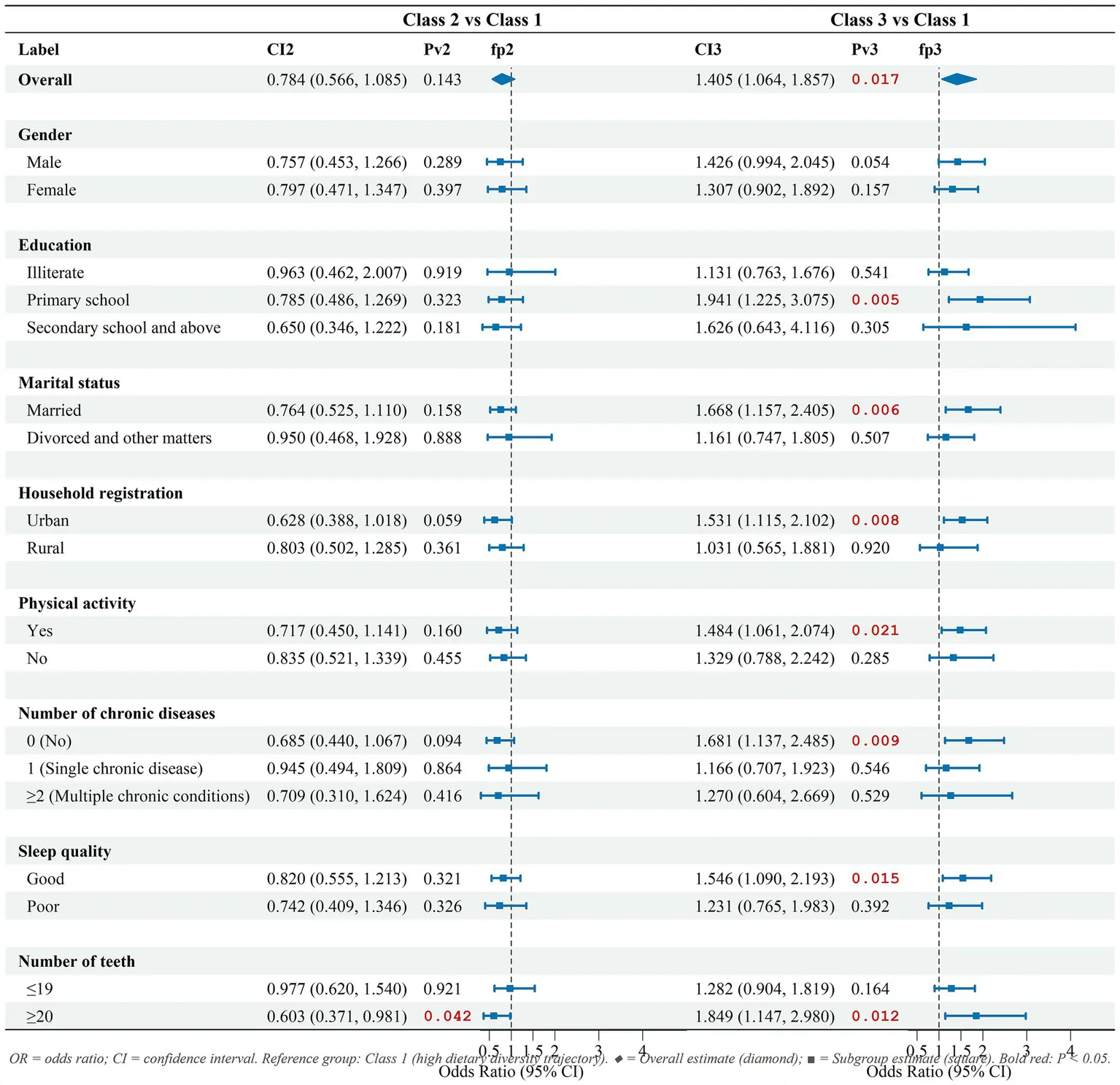
Forest plot for subgroup analyses.

To further investigate potential heterogeneity in the associations between DDS trajectories and depressive symptoms, subgroup analyses were conducted across multiple demographic and health-related strata. The results are shown in Figure 3. Within each subgroup, binary logistic regression models were applied using Class 1 as the reference group, with adjustment for the same covariates as in the primary analysis. Across the overall sample, Class 3 was significantly associated with a higher risk of depressive symptoms (OR = 1.405, 95% CI: 1.064–1.857, *p* = 0.017), and this association was more pronounced among participants with primary school education, married status, urban household registration, engagement in physical activity, absence of chronic diseases, good sleep quality, and ≥20 teeth. The association between Class 2 and depression risk did not reach statistical significance (OR = 0.784, 95% CI: 0.566–1.085, *p* = 0.143), although the direction remained protective. Among participants with ≥20 teeth, both trajectory classes were statistically significant: Class 2 showed a protective effect, whereas Class 3 showed a risk effect.

### 3.6 Dose–response relationship analysis

**Figure 4:**
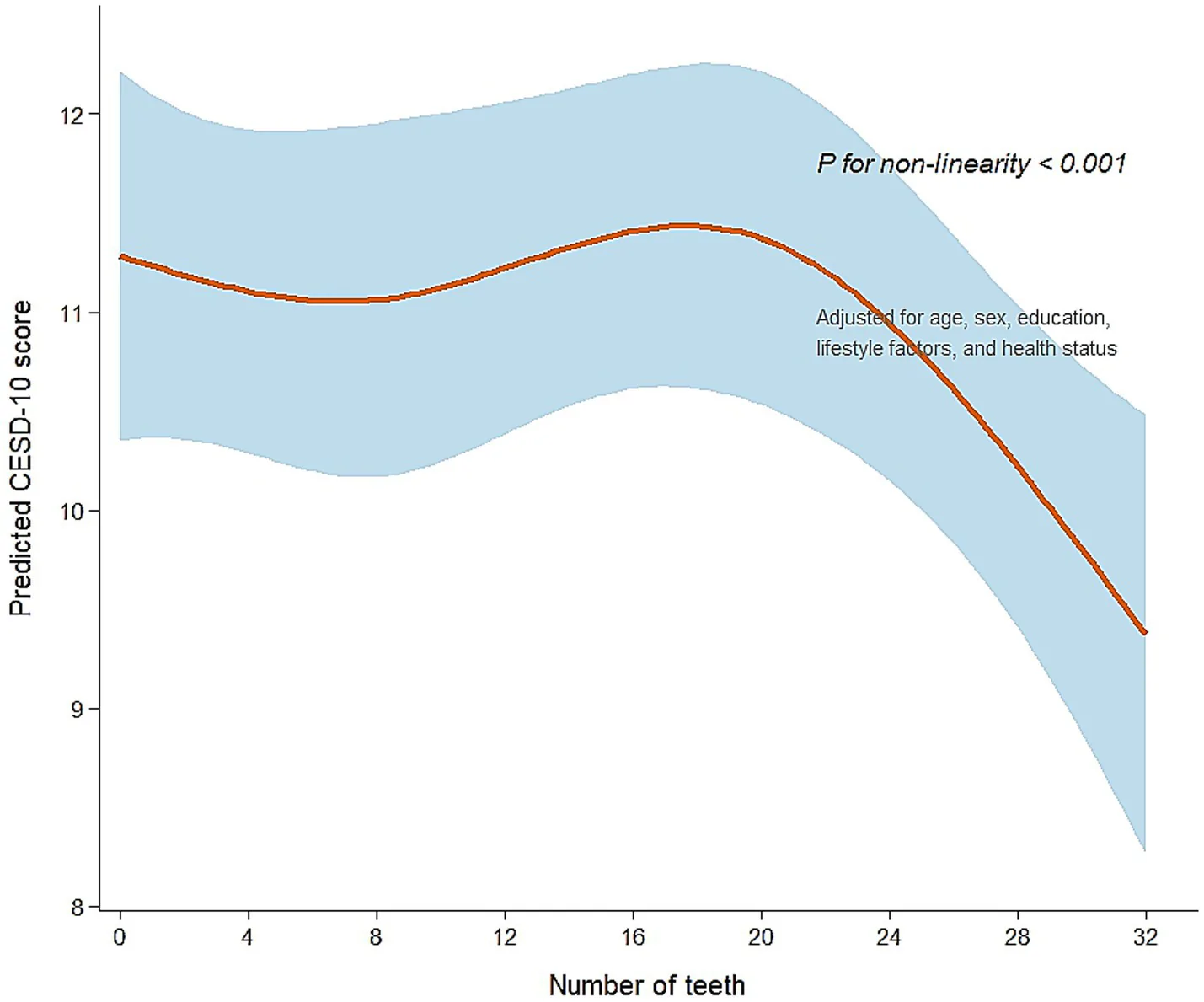
Dose–response curve of number of teeth and depressive scores.

RCS analysis revealed a significant nonlinear association between number of teeth and depressive scores (*P* for nonlinearity < 0.001), characterized by an initial plateau, followed by a slight fluctuation and a subsequent decline. When the number of teeth was at a lower level (0–7 teeth), the curve remained relatively flat. As the number of teeth increased from 8 to 18, CESD-10 scores exhibited a modest fluctuating upward trend, though this fluctuation did not reach statistical significance. When the number of teeth exceeded 20, predicted depressive scores demonstrated a pronounced and sustained declining trend. A greater number of natural teeth was independently and significantly associated with lower depressive scores, and this pattern remained robust after adjustment for multiple confounding factors. The dose–response curve is presented in Figure 4.

## 4 Discussion

This study utilized ten years of longitudinal cohort data from the CLHLS (2008–2018) to evaluate the impact of long-term dietary diversity trajectories on depression among older adults in China. By applying LCGA, three distinct DDS trajectory classes were identified, and their associations with depressive scores were systematically examined. The results showed that the low-increasing trajectory was associated with significantly higher depressive scores, whereas the high-decreasing trajectory was associated with relatively lower depressive scores. These associations remained robust after adjustment for sociodemographic, lifestyle, and health-related factors.

The three DDS trajectories identified in this study differ to some extent from those reported in prior research. Ding et al. (28), using data spanning 2014–2018, identified two DDS trajectories: a “persistent high DDS trajectory” and a “low but slowly rising DDS trajectory.” This discrepancy may be explained by differences in the observation period and sample inclusion criteria between studies. The three trajectories identified in the present study exhibited significant differences in baseline characteristics, consistent with the findings of prior studies on DDS trajectories (21). Class 2 had the highest baseline DDS but experienced a progressive decline over time and comprised a higher proportion of male and rural residents. Class 3 had the lowest baseline DDS but showed a gradual upward trend and included a higher proportion of urban residents, those with relatively higher educational attainment, and the largest proportion of female participants. These observations suggest that older adults from different socioeconomic and demographic backgrounds may follow markedly divergent DDS trajectories, a finding further supported by Lv et al. (34).

The present findings indicate that, compared with the relatively stable dietary diversity of Class 1, Class 3 exhibited significantly higher depressive scores in 2018, while Class 2 demonstrated significantly lower depressive scores. These differences remained statistically significant after adjustment for sociodemographic, lifestyle, and health-related factors, consistent with prior evidence linking lower DDS with elevated depression risk among older adults (19, 20). Ding et al. (28), analyzing CLHLS data from 2014 to 2018, identified two DDS trajectories and found that the low but slowly rising trajectory was associated with a significantly higher risk of depressive symptoms, lending support to the present findings. The current study further refines this conclusion by delineating three trajectory classes over a ten-year observation period, thereby reinforcing the differential impact of distinct dietary trajectories on depressive outcomes. In addition, Zhou et al. (15) demonstrated that adherence to a healthy plant-based dietary pattern was associated with a lower likelihood of unfavorable depressive trajectories among older adults. Although Class 2 had a higher baseline depressive score than Class 1, it still showed significantly lower adjusted depressive scores after controlling for confounders (*B* = −0.902). This suggests that baseline differences may reflect underlying sociodemographic composition rather than dietary diversity itself.

From a biological perspective, the pathways through which dietary diversity influences depression are multifaceted. Dietary diversity is closely related to the composition and diversity of the gut microbiota (35), which influences mood regulation through the gut–brain axis (36). Prolonged low dietary diversity may lead to gut dysbiosis, which in turn activates the hypothalamic–pituitary–adrenal (HPA) axis, a pathway implicated in depressive disorders (37). Gut dysbiosis may also promote the release of pro-inflammatory cytokines, increasing depression risk (38). Furthermore, dietary diversity ensures adequate intake of essential nutrients, including antioxidants, B vitamins, zinc, and magnesium, which are critical for neurotransmitter synthesis and the maintenance of brain function (39, 40). Chronic deficiencies in these nutrients, resulting from sustained low dietary diversity, may exert cumulative detrimental effects on neurological function (41).

In the present study, despite the marked decline in DDS observed in Class 2 over the observation period, participants in this class continued to exhibit relatively lower depressive scores than those in the stable trajectory after multivariable adjustment. One possible explanation is that individuals in the high-decreasing trajectory differed from other groups in their earlier dietary patterns or underlying characteristics, which may be associated with subsequent depressive symptoms. However, this interpretation remains speculative, and alternative explanations cannot be excluded. Residual confounding, socioeconomic differences, healthy survivor bias, and other forms of selection bias may also have contributed to the observed association. Likewise, although participants in Class 3 showed gradual improvements in dietary diversity over time, their higher depressive scores may reflect the cumulative influence of prolonged exposure to relatively low dietary diversity during earlier follow-up, or other unmeasured factors (42, 43). Given that the association for Class 2 was only marginally significant, and that residual confounding and selection bias cannot be fully excluded, these findings should be interpreted as longitudinal associations rather than causal relationships, and further studies are needed to confirm these observations.

Sensitivity analyses, conducted by dichotomizing the 2018 depressive score, confirmed that the significant association between Class 3 and elevated depression risk remained robust (OR = 1.405, 95% CI: 1.064–1.857), fully consistent with the primary analysis. The protective direction of the association between Class 2 and depression was maintained, though it did not reach statistical significance, which may be related to reduced statistical power in this subgroup. Additionally, compared with participants without chronic diseases, those with ≥2 chronic conditions were at significantly higher risk of depressive symptoms. Subgroup analyses further suggested that the association between Class 3 and elevated depression risk was more pronounced among participants with primary school education, married status, urban household registration, physical activity participation, absence of chronic diseases, good sleep quality, and ≥20 teeth. Notably, in the subgroup of participants with ≥20 teeth, both trajectory classes exhibited statistically significant associations, with Class 2 being associated with a lower risk of depressive symptoms and Class 3 being associated with a higher risk of depressive symptoms. One possible explanation is that among individuals with better dental function and fewer dietary intake barriers, differences in DDS trajectories may be more readily reflected in depressive outcomes. Nevertheless, these subgroup analyses were exploratory and were conducted primarily to assess the consistency of the observed associations across different participant characteristics. Formal interaction tests and multiple testing corrections were not performed. Therefore, the subgroup findings should be interpreted with caution, as some observed associations may have occurred by chance and require confirmation in future studies.

Furthermore, RCS analysis revealed a significant nonlinear association between number of teeth and depressive scores, characterized by an initial plateau, a brief transient fluctuation, and a sustained decline at ≥20 teeth. Prior research has indicated that retaining at least 20 natural teeth represents the standard for maintaining acceptable masticatory and aesthetic function (44). Accordingly, fewer than 20 teeth may signify entry into a phase of functionally limiting tooth loss (45). Reduced masticatory capacity may subsequently limit dietary diversity and increase psychosocial burden, thereby contributing to depression risk (46). These findings are further supported by Wang and Sun (22), who reported that moderate tooth loss (<20 teeth) was more strongly associated with depressive symptoms than mild tooth loss (≥20 teeth).

Several strengths of this study merit acknowledgment. First, this study leverages a large-scale, nationally representative longitudinal cohort dataset. The use of trajectory analysis overcomes the limitations of cross-sectional designs by capturing dynamic changes in dietary diversity over time, effectively reflecting the long-term cumulative effects and heterogeneity of dietary behavior, thereby enabling more precise identification of high-risk subpopulations and enhancing the generalizability of the findings. Second, the robustness of the primary findings was further substantiated by sensitivity analyses and subgroup analyses across multiple participant characteristics, with adjustment for a comprehensive range of sociodemographic and health-related variables. Finally, the application of RCS analysis to characterize the dose–response relationship between number of teeth and depressive scores addresses the limitations of conventional linear regression in detecting threshold effects, contributing to a more comprehensive understanding of the interrelationships among oral health, nutritional intake, and mental health in older adults.

This study is not without limitations. First, although longitudinal data from multiple waves were used to construct DDS trajectories, the depressive outcome was measured exclusively at the endpoint wave (2018), precluding a dynamic longitudinal examination of the evolving relationship between DDS trajectories and depressive symptoms over time. Future studies incorporating repeated depressive assessments at each follow-up wave would better elucidate the temporal dynamics of this relationship. Second, DDS was derived from a self-reported food frequency questionnaire, which is subject to recall bias and does not capture the quantity of food intake, a limitation common to nutritional epidemiology research (21).

Third, despite adjustment for multiple important covariates, residual confounding cannot be entirely excluded given the observational nature of the study (47). Moreover, some covariates included in the regression models may have changed during the 10-year follow-up period. Although baseline characteristics were adjusted in the present analyses, the potential influence of time-varying changes in these covariates could not be fully evaluated. Although the fully adjusted model explained a relatively modest proportion of the variance in depressive scores (R^2^ = 0.086), this finding is not unexpected given the multifactorial nature of depression in older adults. Depression is influenced by a broad range of biological, psychological, social, and environmental determinants. In addition, both dietary diversity and depressive symptoms were assessed using questionnaire-based measures, which may introduce measurement error. Consequently, the modest explained variance is more likely attributable to unmeasured determinants of depression and measurement limitations rather than deficiencies in model specification. Accordingly, the present findings should be interpreted as evidence of longitudinal associations rather than strong predictive ability.

Fourth, compared with excluded participants, individuals included in the final analysis were generally younger, healthier, and had more favorable socioeconomic characteristics at baseline. This pattern is consistent with the healthy survivor effect commonly observed in long-term aging cohort studies, whereby participants with poorer health are more likely to die, be lost to follow-up, or have incomplete data over time. Although all relevant baseline characteristics were included as covariates in the multivariable models, residual selection bias and survivor bias cannot be completely ruled out. Therefore, caution is warranted when generalizing these findings to frailer older populations.

Finally, although participants with baseline depressive symptoms were excluded, reverse causality cannot be completely ruled out because the final DDS assessment and depressive outcome were both measured in 2018. Individuals with depressive symptoms may experience changes in appetite, food preferences, or eating behaviors that subsequently influence dietary diversity. In addition, baseline depressive symptoms were identified using the 5-item depression scale available in the 2008 CLHLS, whereas the study outcome was assessed using the CESD-10 in 2018. Although both instruments have been used to evaluate depressive symptoms in older Chinese populations, the use of different assessment tools may have introduced some inconsistency in identifying depressive symptoms across study waves. Despite the use of repeated dietary assessments over the 10-year follow-up, the observational design precludes definitive causal inference. Taken together, these limitations indicate that the observed associations should not be interpreted as evidence of causality. Moreover, because the study population consisted exclusively of Chinese older adults, caution is warranted when generalizing these findings to populations with different cultural or dietary backgrounds.

## 5 Conclusion

Based on ten years of longitudinal cohort data, this study identified three distinct DDS trajectory classes among older adults in China: stable, high-decreasing, and low-increasing. Compared with the stable trajectory, the low-increasing trajectory was independently associated with higher depressive scores, whereas the high-decreasing trajectory showed an inverse association with depressive scores. A significant nonlinear dose–response relationship was observed between number of teeth and depressive scores, with retaining ≥20 natural teeth being associated with lower depressive scores. These findings suggest that maintaining a relatively higher level of dietary diversity over the long term may contribute to better mental health among older adults, and that early intervention, promoting dietary diversification at the onset of aging, represents a critical window of opportunity for achieving this goal. Future research should prioritize the development and evaluation of early dietary intervention strategies targeting older adult populations, and further investigate the dynamic temporal relationships among dietary diversity, oral health, and depression, with a view to more comprehensively elucidating the underlying mechanisms linking these factors.

## Data Availability

The original contributions presented in the study are included in the article/Supplementary material, further inquiries can be directed to the corresponding author/s.
