# Supplementary material for: Associations of dietary diversity trajectories with depressive symptoms among Chinese older adults: a 10-year longitudinal study
Source: Front Nutr. 2026 Aug 21;13:1873667. doi: 10.3389/fnut.2026.1873667 (PMC13541433; doi:10.3389/fnut.2026.1873667)
Supplement: Supplementary file 1 [file Supplementary_file_1.docx]

***Supplementary Material***

**Associations of Dietary Diversity Trajectories with Depressive Symptoms among Chinese Older Adults: A 10-year longitudinal study**

**Table S1: Model selection statistics for one- to five-class latent class growth models**

**Table S2: Multicollinearity diagnostics for the multivariable linear regression models**

**Table S3: Diagnostic tests for the multivariable linear regression models (Model 1–3)**

**Table S4: Sensitivity analysis (Binary logistic analysis)**

**Table S5:** **Comparison of included and excluded participants**

**Table S6: Results of validity analysis for the CESD-10**

**Table S7: Rotated factor loading matrix for the CESD-10**

**Figure S1: Q-Q plot**

**Table** **S1: Model selection statistics for one- to five-class latent class growth models**

| **Number of classes** | **AIC** | **BIC** | **Entropy** | **Average posterior probability** | **Smallest class (%)** |
| --- | --- | --- | --- | --- | --- |
| 1 | -28730.47 | -28775.86 | — | — | 100 |
| 2 | -28701.72 | -28757.87 | 0.75 | 0.880 / 0.868 | 721 (46.9%) |
| 3 | -28660.65 | -28727.56 | 0.78 | 0.815/ 0.779 / 0.767 | 205 (13.3%) |
| 4 | -28668.76 | -28746.42 | 0.74 | 0.695 / 0.771 / 0.682 / 0.821 | 239 (15.5%) |
| 5 | -28681.55 | -28769.98 | 0.72 | 0.585 / 0.746 / 0.574 / 0.783 / 0.617 | 121 (7.9%) |

The three-class model was selected based on the overall consideration of statistical fit (AIC, BIC, entropy, and average posterior probabilities), minimum class size (>5%), and clinical interpretability.

**Table S2: Multicollinearity diagnostics for the multivariable linear regression models**

| **Variable** | **Tolerance** | **VIF** |
| --- | --- | --- |
| Sex | 0.90 | 1.11 |
| Residence | 0.86 | 1.16 |
| Education | 0.81 | 1.24 |
| Marital status | 0.88 | 1.14 |
| Smoking | 0.80 | 1.24 |
| Drinking | 0.86 | 1.16 |
| Exercise | 0.87 | 1.15 |
| Sleep quality | 0.97 | 1.03 |

All VIF values were < 5, indicating no evidence of multicollinearity.

**Table S3: Diagnostic tests for the multivariable linear regression models (Model 1-3)**

| **Diagnostic test** | **Model 1** | **Model 2** | **Model 3** | **Interpretation** |
| --- | --- | --- | --- | --- |
| Model F (*P* value) | 11.187 (<0.001) | 8.439 (<0.001) | 8.889 (<0.001) | All models significant |
| Multicollinearity (VIF range) | — | 1.00–1.16 | 1.00–1.24 | All VIF < 5, no collinearity |
| Standardized residuals: range | — | — | -2.56 to 3.61 | Mostly within ±3 |
| Standardized residuals: mean (SD) | — | — | 0.00 (0.995) | Approximately normal |

**Table S4: Sensitivity analysis (Binary logistic analysis)**

| **Variables** | **B** | **SE** | **95% CI** | ***P-*value** |
| --- | --- | --- | --- | --- |
| Class2 | -0.243 | 0.166 | (0.566, 1.085) | 0.143 |
| Class3 | 0.340 | 0.142 | (1.064, 1.857) | 0.017 |
| Male (Female) | 0.141 | 0.141 | (0.873, 1.518) | 0.319 |
| Urban (Rural) | 0.014 | 0.129 | (0.787, 0.306) | 0.916 |
| Divorced / Widowed (Married) | 0.179 | 0.129 | (0.929, 1.538) | 0.165 |
| Unmarried (Married) | 0.697 | 0.657 | (0.553, 7.280) | 0.289 |
| Primary school (Illiterate) | -0.292 | 0.130 | (0.579, 0.963) | 0.025 |
| Secondary school and above (Illiterate) | -0.647 | 0.179 | (0.368, 0.743) | ＜0.001 |
| Alcohol consumption (No) | 0.008 | 0.141 | 0.765, 1.329 | 0.955 |
| Physical activity (No) | -0.192 | 0.122 | (0.650, 1.049) | 0.116 |
| Smoking (No) | -0.263 | 0.145 | (0.579, 1.021) | 0.069 |
| Number of teeth | -0.014 | 0.005 | (0.976, 0.996) | 0.008 |
| Quality of life score | 0.085 | 0.073 | (0.943, 1.258) | 0.244 |
| 1 chronic disease (0) | 0.228 | 0.128 | (0.977, 1.613) | 0.075 |
| >2 chronic diseases (0) | 0.327 | 0.161 | (1.011, 1.900) | 0.042 |
| Good sleep (Poor sleep) | -0.350 | 0.123 | (0.554, 0.897) | 0.004 |

**Table S5:** **Comparison of included and excluded participants**

| **Characteristics** | **Included (*N*=1,538)** | **Excluded (*N*=15,416)** | ***P*-value** |
| --- | --- | --- | --- |
| Age (years), mean ± SD | 73.95 ± 7.16 | 88.14 ± 11.58 | <0.001 |
| Gender, *n* (%) |  |  | <0.001 |
| Male | 759 (49.3%) | 6,493 (42.1%) |  |
| Female | 779 (50.7%) | 8,923 (57.9%) |  |
| Household registration, *n* (%) |  |  | <0.001 |
| Urban | 492 (32.0%) | 6,169 (40.0%) |  |
| Rural | 1,046 (68.0%) | 9,247 (60.0%) |  |
| Education, *n* (%) |  |  | <0.001 |
| Illiterate | 670 (43.6%) | 9,867 (64.0%) |  |
| Primary school | 628 (40.8%) | 4,041 (26.2%) |  |
| Secondary school and above | 240 (15.6%) | 1,508 (9.8%) |  |
| Marital status, *n* (%) |  |  | <0.001 |
| Married | 1,008 (65.5%) | 4,527 (29.4%) |  |
| Divorced/widowed | 515 (33.5%) | 10,750 (69.7%) |  |
| Never married | 15 (1.0%) | 139 (0.9%) |  |
| Smoking, *n* (%) |  |  | <0.001 |
| Yes | 369 (24.0%) | 2,597 (16.8%) |  |
| No | 1,169 (76.0%) | 12,819 (83.2%) |  |
| Alcohol consumption, *n* (%) |  |  | <0.001 |
| Yes | 363 (23.6%) | 2,570 (16.7%) |  |
| No | 1,175 (76.4%) | 12,846 (83.3%) |  |
| Physical activity, *n* (%) |  |  | <0.001 |
| Yes | 569 (37.0%) | 4,076 (26.4%) |  |
| No | 969 (63.0%) | 11,339 (73.6%) |  |
| Sleep quality, *n* (%) |  |  | <0.001 |
| Good | 986 (64.1%) | 13,848 (89.8%) |  |
| Poor | 552 (35.9%) | 1,568 (10.2%) |  |
| Number of chronic diseases, *n* (%) |  |  | 0.352 |
| 0 (No) | 797 (51.8%) | 7,831 (50.8%) |  |
| 1 (Single chronic disease) | 478 (31.1%) | 4,718 (30.6%) |  |
| ≥2(Multiple chronic conditions) | 263 (17.1%) | 2,867 (18.6%) |  |
| Number of teeth, mean ± SD | 14.27 ± 10.54 | 7.46 ± 9.96 | <0.001 |
| Quality of life score, mean ± SD | 2.37 ± 0.80 | 3.11 ± 2.06 | <0.001 |
| DDS total score, mean ± SD | 5.92 ± 2.63 | 5.54 ± 2.53 | <0.001 |

*P*< 0.05 indicates statistically significant difference among the three groups.

**Table S6: Results of validity analysis for the CESD-10**

| **Measure** | **Value** |
| --- | --- |
| KMO measure of sampling adequacy | 0.888 |
| Bartlett's test of sphericity | χ² = 4360.402, df = 45, P < 0.001 |
| Number of factors extracted (eigenvalue > 1) | 2 |
| Total variance explained | 52.33% |

**Table S7: Rotated factor loading matrix for the CESD-10**

| **Item** | **Factor 1** | **Factor 2** |
| --- | --- | --- |
| CESD1 | 0.686 | -0.257 |
| CESD 2 | 0.569 | -0.330 |
| CESD 3 | 0.759 | -0.217 |
| CESD 4 | 0.662 | -0.221 |
| CESD 5 | 0.526 | 0.630 |
| CESD 6 | 0.700 | -0.165 |
| CESD 7 | 0.557 | 0.517 |
| CESD 8 | 0.705 | -0.035 |
| CESD 9 | 0.721 | 0.018 |
| CESD 10 | 0.442 | 0.413 |
| Eigenvalue | 4.098 | 1.135 |
| % of variance | 40.98% | 11.35% |
| Cumulative % | 40.98% | 52.33% |

**Figure S1: Q-Q plot**
